# Fabrication of CA/TPU Helical Nanofibers and its Mechanism Analysis

**DOI:** 10.1186/s11671-018-2516-3

**Published:** 2018-04-16

**Authors:** Huihui Wu, Shihang Zhao, Lei Han

**Affiliations:** 1 Pan Tianshou Arts and Design Academy, Ningbo University, Ningbo, Zhejiang Province 315211 People’s Republic of China; 2 The School of Materials Science and Chemical Engineering, Ningbo University, Ningbo, Zhejiang Province 315211 People’s Republic of China

**Keywords:** Fabrication, Interfacial interaction, Helical nanofibers, Mechanism

## Abstract

To explore the mechanism of cellulose acetate (CA)/thermoplastic polyurethane (TPU) on the fabrication of helical nanofibers, a series of experiments were conducted to find the optimum spinning conditions. The experimental results show that the CA (14 wt%, DMAc/acetone, 1/2 volume ratio)/TPU2 (18 wt%, DMAc/acetone, 3/1 volume ratio) system can fabricate helical nanofibers effectively via co-electrospinning. We focus on the interfacial interaction between the polymer components induced by the polymer structure and intrinsic properties, including solution properties, hydrogen bonding, and miscibility behavior of the two solutions. Differential scanning calorimetry (DSC) and Fourier transform infrared spectroscopy (FTIR) are employed to investigate the interfacial interaction between the two phases of the polymer system. The analysis results provide the explanation of the experimental results that the CA/TPU system has the potential for producing helical nanofibers effectively. This study based on the interfacial interaction between polymer components provides an insight into the mechanism of CA/TPU helical fiber formation and introduces a richer choice of materials for the application of helical fibers.

## Background

Helical structures with broad spectrum of applications in the fields of nanoscale sensors, filtration materials, oil sorbents, solar cells, and so on [1, 2] have attracted extensive attention due to their large surface-area-to-volume ratio and high porosity. The introduction of helical structure into micro/nanofibers can improve fiber resilience and flexibility, and this three-dimensional (3D) structure of the helices can provide the fiber mat larger porosity [3]. Helical structures can be found in many natural systems such as plant tendrils and fine wool, which are regarded as the consequence of different shrinkages (or extensions) and results in forced winding of the structure [4]. Zhang et al. [5] focused on the formation, structure, and function of the most common chiral nanoarchitectures and explored how the molecules can form hierarchical chiral nanoarchitectures. The mechanism of such an asymmetric deformation should also be used for generating fiber curvature. Co-electrospinning, compared with the other methods, such as chemical vapor deposition [6], sol–gel [7], and hydrothermal [8], is a simple and efficient method for generating composite fibers with kinds of morphologies at the micro- and nanoscales.

With the aid of co-electrospinning technique, several researchers successfully prepared three-dimensional helical nanofibers from two component solutions. Lin et al. [9] obtained nanoscale biomimetic wool fibers by electrospinning PAN and TPU using a side-by-side co-electrospinning arrangement. Chen et al. [10] utilized three kinds of co-electrospinning spinnerets to produce nanosprings from PU and Nomex. Using side-by-side electrospinning, Zhang et al. [11] reported the generation of fibers with curled and helical morphologies from poly(ethylene glycol terephthalate) (HSPET) and poly(ethylene propanediol terephthalate) (PTT). In the above researches, the helical nanofibers obtained are described as three-dimensional and spring-like structures with nano- to microscale helix diameters. The authors attributed the generation of helical fibers to the fact that the two components involved in co-electrospinning display different shrinkages after electrospinning. But there is no detailed analysis and explanation of the formation mechanism of helical fibers. Based on the concept that an elastomeric and a stiff polymer in co-electrospinning may introduce longitudinal stress and result in coiled shapes of the bicomponent fibers, our previous studies [12] reported the fabrication of helical nanofibers via co-electrospinning. We compared three component systems, Nomex/TPU, PAN/TPU, and PS/TPU, which represent three kinds of polymer composition arrangements in co-electrospinning, and explored the role of polymer chain rigidity, miscibility, and hydrogen bonding on the formation of helical fibers. It has been experimentally verified that Nomex/TPU system can form fine helical fibers. However, Nomex is a non-hydrophilic polymer, which limited its application in biological tissue and adsorption filtration [13].

Therefore, in this article, based on the previous research, we further discuss the CA/TPU co-electrospinning conditions and analyze its mechanism of helical fiber formation. We prepare the composite helical nanofibers with CA, the rigid component and TPU, and the elastomeric component by co-electrospinning technique. In the experimental part, we conducted single-spinning experiments of CA and TPU, respectively. Different CA solution concentration and solvent systems (volume ratio of DMAc to acetone) were applied to find the processing conditions of fine CA fibers. And in the TPU spinning system, we tried two solvent systems, TPU1 (DMAc/THF, 3/1 volume ratio) and TPU2 (DMAc/acetone, 3/1 volume ratio), which enable lower interfacial tension with CA solution. Then, CA with different LiCl concentration and TPU of different solvent systems were conducted to do co-electrospinning experiments, respectively. In the discussion section, we focus on the interfacial interaction between CA and TPU components induced by different polymer structure and intrinsic properties, including solution properties, miscibility, and hydrogen bonding of the two solutions. Thermal and spectroscopic techniques including DSC and FTIR are utilized to study the interaction behavior of the CA/TPU pair. This study provides the insight into the CA/TPU helical fiber formation and introduces a richer choice of materials for the application of helical fibers.

## Experimental

### Materials

Cellulose acetate (CA, white powder, *M*_W_ = 100 W g/mol) was purchased from Acros Organics. Thermoplastic polyurethane (TPU, Desmopan DP 2590A) was from Bayer Materials Science. *N*, *N*-dimethylacetamide (DMAc, 0.938–0.942 g/ml at 20 °C, surface tension 25.3 dyne/cm, vapor pressure 0.17 kPa (20 °C)), acetone (0.788 g/ml at 20 °C, surface tension 18.8 dyne/cm, vapor pressure 24.64 kPa (20 °C)), tetrahydrofuran (THF, 0.887–0.889 g/ml at 20 °C, surface tension 28.8 dyne/cm, vapor pressure 18.9 kPa (20 °C)), and lithium chloride anhydrous (LiCl, *M*w = 42.39 g/mol) were all purchased from Shanghai Chemical Reagents Co., Ltd., China. All of these materials were used without further purification. All experiments were performed at about 25 °C and 40%~ 50% RH.

### Co-electrospinning

**Fig. 1:**
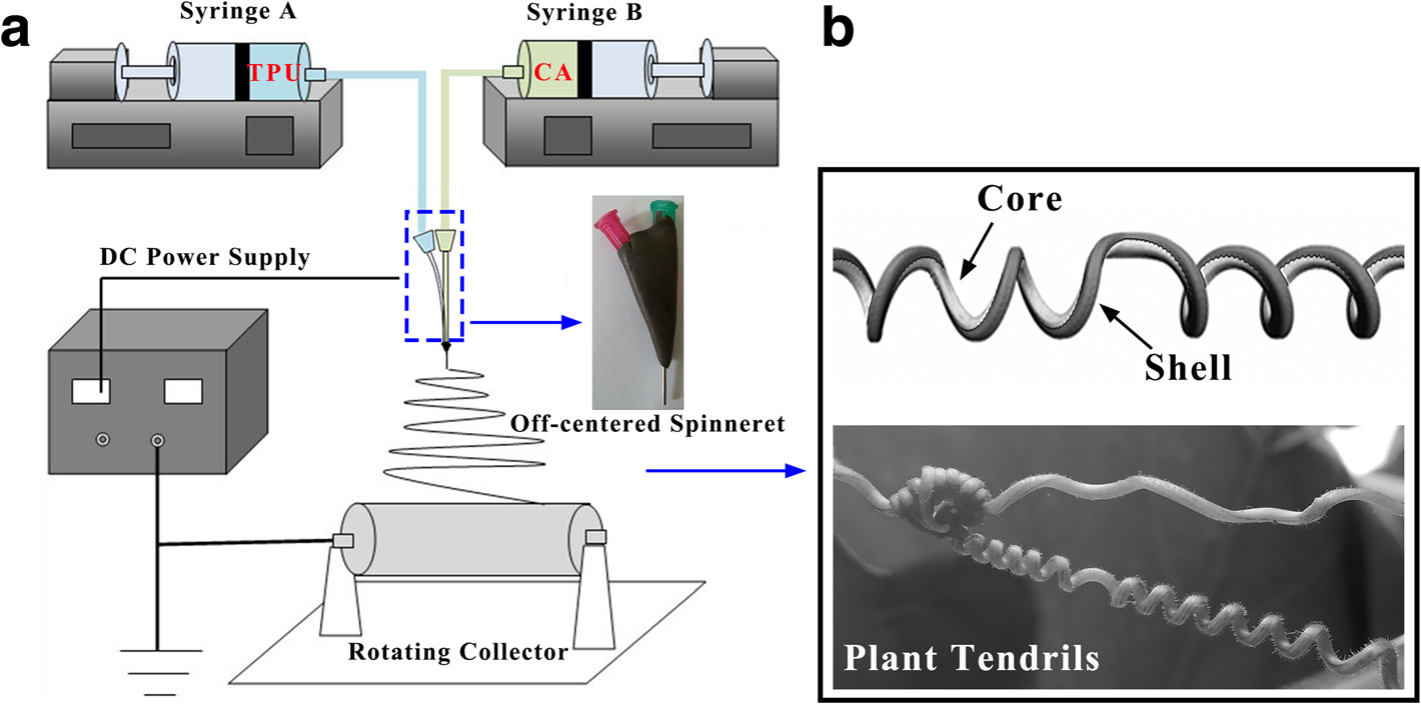
**a** Schematic of the off-centered co-electrospinning system. **b** Formation mechanism of helical nanofibers

CA solution with different CA and LiCl concentration was prepared by dissolving CA powder and LiCl in the mixture solvents of DMAc and acetone. TPU solution with 18 wt% concentration was prepared by dissolving TPU pellets in mixture solvents of DMAc/THF (3/1 volume ratio), referred to as TPU1 and in mixture solvents of DMAc/acetone (3/1 volume ratio), referred to as TPU2. All solutions were stirred for 5 h at ambient temperature and set aside overnight for preparation. As shown in Fig. 1a, a co-electrospinning system was used to eject the core and shell polymer solutions through an off-centered spinneret via corresponding syringes and pumps. A high-voltage supply was applied to the spinneret and the rotating collector with the linear velocity of 14.24 cm/s. Figure 1b show the formation mechanism of helical nanofibers: the core component involved in the nanofiber display greater shrinkage than the shell component, like plant tendrils.

### Characterizations

#### Fiber Morphology

The morphology of the resultant core-shell fibers were observed under a Scanning Electron Microscope (SEM) (JSM-5600LV, Japan) after gold coating.

#### DSC

The glass transition temperatures of the blends were performed using a DSC from DSC-4000 in a nitrogen atmosphere with temperature. The measurement was made using 5–10 mg sample on a DSC sample cell after the sample was quickly cooled to − 80 °C from the melt of the first scan. The glass transition temperature was obtained as the inflection point of the jump heat capacity with scan rate of 10 °C/min and temperature range of − 80~300 °C.

#### FTIR

Infrared spectra were recorded on a Bruker Vector 33 FTIR spectrophotometer, and 32 scans were collected with a spectral resolution 1 cm^−1^. The film used in this study was sufficiently thin to obey the Beer-Lambert law. IR spectra recorded at elevated temperatures were obtained by using a cell mounted inside the temperature-controlled compartment of the spectrometer.

The solution properties are shown in Table 1. Blends with different component pairs were prepared by blending solutions. Blends were stirred for 8 h and were allowed to evaporate slowly at room temperature for 2 days. Films of the blends were then dried at 90 °C for 1 day to ensure total elimination of solvents.

**Table 1:** Properties of different electrospinning solutions

	Solvent (volume ratio)	Concentration (wt.−%)	Amount of LiCl (wt.−%)	Surface tension (N m^−1^)	Viscosity (Pa.s)	Conductivity (μS cm^−1^)
CA	DMAc: acetone = 1:2	12	0	29.76	1.211	–
		14	0	30.12	1.263	–
		14	0.5	31.49	1.347	1.19
		14	1	35.84	1.373	1.54
		14	2	38.21	1.824	1.93
		16	0	37.89	2.137	–
TPU1	DMAc: THF = 3:1	18	–	34.45	1.462	–
TPU2	DMAc: acetone = 3:1	18	–	25.34	1.321	–

### Experimental Results

To explore the mechanism of CA/TPU helical fibers and the role of solvent effects, we designed two part experiments: the first part was carried out to select the suitable single spinning parameters, and in the second part, the combinatorial experiment: two systems of polymer composition, CA/TPU1 and CA/TPU2, were studied.

**Fig. 2:**
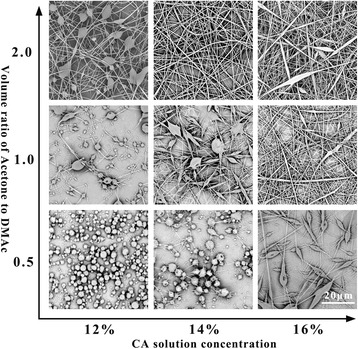
SEM images of CA single electrospinning experiments of *x*-axis: CA solution concentration, of *y*-axis: volume ratio of DMAc to acetone. The applied voltage is 15 kV, the working distance is 10 cm, and the flow rate is 0.15 ml/h

Figure 2 shows the results of the single CA electrospinning experiments with different solution concentration and solvent systems under the processing conditions of 15 kV applied voltage, 10 cm working distance, and 0.2 ml/h flow rate. The *x*-axis shows CA solution concentration, and *y*-axis denotes the volume ratio of DMAc to acetone. We found that under the same CA solution concentration, with the increase of acetone proportion in the CA solution, the less beads formed on the CA nanofibers. However, during the experiment process, the CA will form coagulum easily appeared on the needle tip, which resulted to an uneven fiber fineness because the vapor pressure (about 24.64 kPa (20 °C)) of acetone is too high. As the CA concentration increases, the spinning balls turn to homogeneous fibers, but when the concentration is too high, some spindles begin to appear on the fibers. In consideration of the relative stable spinning process, we chose CA solution concentration of 14 wt% dissolved in the volume ratio of acetone to DMAc of 2. Another note that should be pointed out is, in order to meet the demands of helical fiber spinning later, when we added LiCl in CA solution, the single spinning fibers emerge as bundles and the spinning process cannot be carried out due to the high conductivity.

**Fig. 3:**
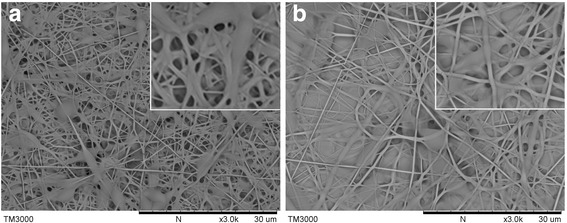
SEM images of the single electrospinning experiments from **a** 18 wt% TPU1 in DMAc/THF of 3/1 volume ratio **b** 18 wt% TPU2 in DMAc/acetone of 3/1 volume ratio. The applied voltage is 15 kV, the working distance is 10 cm, and the flow rate is 0.15 ml/h

Figure 3 shows the results of the single electrospinning experiments of TPU1 and TPU2. As we all know, in sufficiently strong electric fields, jetting sets in at the tip of compound droplets, in which case the entrainment of the core fluid results in the formation of compound fibers [14]. So, in the co-electrospinning, the shell solution acts as a protective layer and surrounds the core layer. Accordingly, the electrospinnable shell solution is critical for the bilayer structure formation, while it appears that the requirements for the spinnability of the core layer by itself are not as critical as the shell layers. In this study, we tried a variety of TPU solution concentrations in the experimental process. Due to the TPU solution as the core layer with low spinnability request, and the following suitable co-spinning required, here, we only show the concentration of 18 wt% TPU pictures for reference. In our previous study [15], we used DMAc: THF = 3:1 as TPU1 solvent to spin helical fibers, which is shown in Fig. 3a. It can be seen that there is a lot of beads on the fibers, although the basic fiber morphology could be distinguished easily. In this study, we used DMAc: acetone = 3:1 as the solvent of TPU2 as a comparison. Figure 3b shows the single spinning of TPU2; as we can see, there is a serious fiber adhesion between layers and almost no fibers formed.

In the next part, we will co-spin CA added with different content of LiCl and TPU (included TPU1 and TPU2), respectively. So, two component systems, CA/TPU1 and CA/TPU2, were chosen in the co-electrospinning. Although the single-spinning TPU results are not satisfactory, as the core layer of the co-spinning, it will show another situation.

**Fig. 4:**
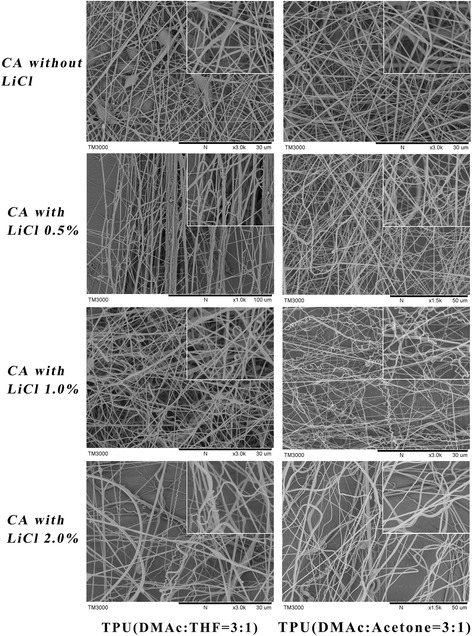
SEM images of two component systems of CA/TPU1 and CA/TPU2, in which the LiCl added in CA solution is from zero to 2 wt%. The processing condition is of 20 kV applied voltage, 15 cm working distance, and 0.15 ml/h flow rate for both component solutions

Figure 4 shows the results of 14 wt% CA dissolved by volume ratio of DMAc to acetone of 0.5, with different LiCl concentration as the shell layer and the two TPU solutions as the core layer. As we can see, when there is no LiCl added in the CA solution, there is no helical fibers formed in both TPU combinations. The CA/TPU1 fibers even contain some beads, while the CA/TPU2 fiber is relatively uniform, with no beads or adhesion appeared between the fiber layers. With the increase of LiCl concentration in CA, both TPU systems begin to appear some helical fibers. When the LiCl concentration is at a low level (0.5 wt%), the CA/TPU1 co-spinning fibers show like bundles with ununiform diameters. As the LiCl concentration increased, the bundling phenomenon disappeared, but there is still not so many helical fibers appeared. When the LiCl concentration reached 2 wt%, the CA/TPU1 fibers show a bit of helical fibers, but due to the high conductivity of the solution, the fiber fineness is not so uniform. By contrast, the CA/TPU2 performance is much better. When the LiCl concentration is 0.5 wt%, the CA/TPU2 fibers become to be bended from straight fiber. As the LiCl concentration reached 1 wt%, considerable amount of helical fibers are observed in the CA/TPU2 fiber web. When the LiCl concentration increased to 2 wt%, the helical fibers have been stretched due to the excessive conductivity of the solution.

We have tried various processing conditions for the two component systems, and the experiments show the similar results that the CA/TPU2 fibers can fabricate helical structures more effectively comparing with the CA/TPU1 system. Only a few fibers show helical structures in the CA/TPU1 fiber web. These experiments demonstrate that the LiCl concentration and solvent systems play a crucial role in the generation of helical fibers. In this study, we furtherly analyze the experimental results through the below three aspects to explain the mechanism of the helical fiber formation.

## Results and Discussion

In this paper, we try to explore the CA/TPU helical fiber spinning mechanism and discuss how the solution properties, miscibility, and hydrogen bonding of the two solutions affect the morphology of the resultant fibers.

### Mechanism of CA/TPU Helical Fibers

**Fig. 5:**
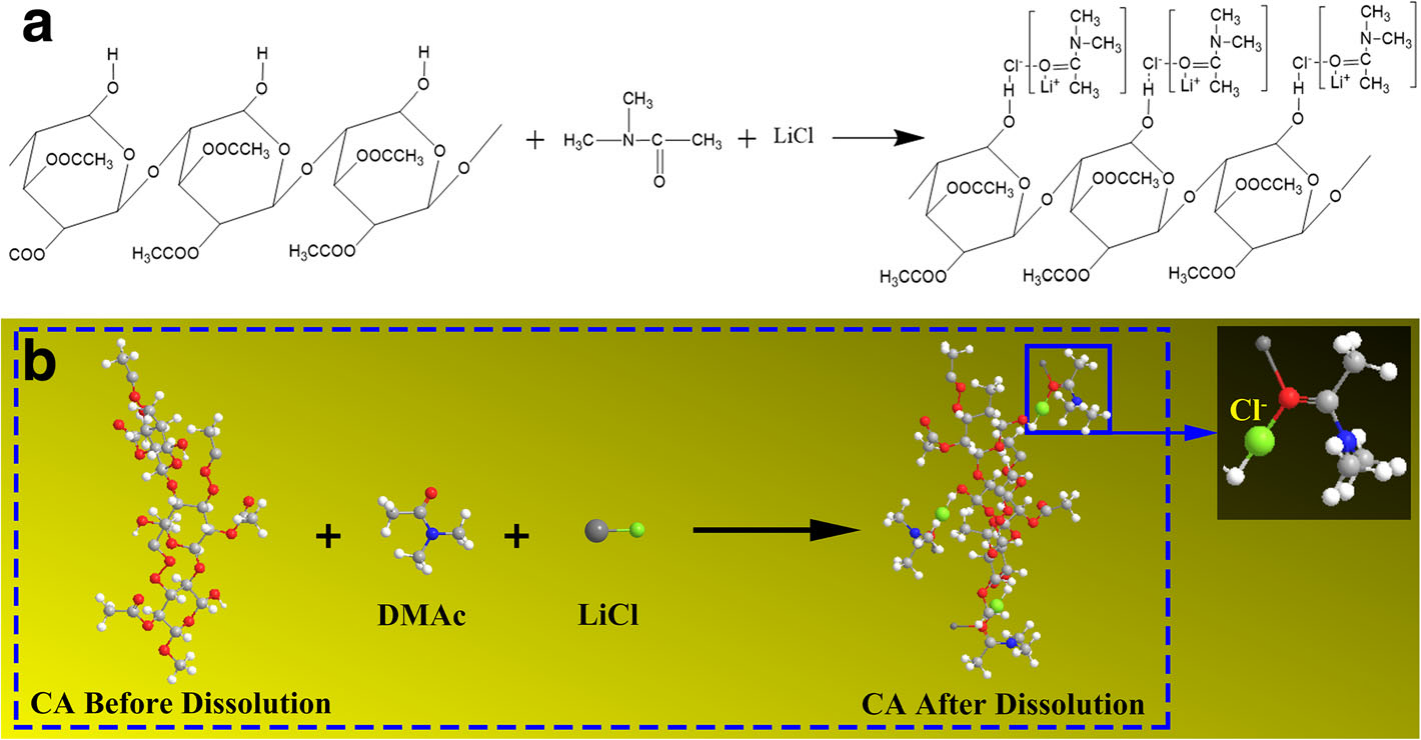
Proposed mechanism for the dissolution of CA in the DMAc/LiCl solvent system: **a** molecular formula and **b** 3D molecular structure

Some researchers have reported CA solubility in LiCl/DMAc solvent system [16, 17]. The mechanism we believe to be operative for CA dissolution is shown in Fig. 5a. The lithium-ion is associated with DMAc to form macrocation complex structure. The chloride ions are associated with the hydroxyl hydrogens in CA by hydrogen bonds. Consequently, it can be found that after dissolution, the negatively charged chloride ions are combined with the polymer chains of CA. This can be used to illustrate the phenomenon of Fig. 4. When without LiCl in CA solution, there is no helical fiber formed, but with the increase of LiCl concentration, the CA/TPU system could form helical fibers. Here, the additive LiCl does not only increase the conductivity of the solution, but also make the CA chain can be effectively stretched due to the negatively charged chloride ions [18]. The stretched orientation of molecular chain is beneficial to increase the rigidity of the hard chain segment, which increases the stiffness difference in the soft segment, and further be beneficial to the formation of helical fibers. As shown in Fig. 5b, in the CA solution, the attraction force generated between the positive charges at the solution surface and negative charges carried by chloride ions in the CA chain helps the formation of the CA/TPU composite jet and is believed to be beneficial to the co-electrospinning process.

### Solution Properties

As we all know, the solution parameters of co-spinning include the solution viscosity, solvent vapor pressure, interfacial tension, and solution conductivity. As shown in Fig. 2, when we change the solvent THF with acetone in TPU, the fiber adhesion phenomenon is reduced. It should be noted that the solvents used by different kinds of TPU are very important. The solution properties are shown in Table 1. As been shown, the solvents of TPU1 are DMAc and THF (3/1 volume ratio), while the solvents of TPU2 are DMAc and acetone (3/1 volume ratio), which result to the different solution properties. As we can see, the surface tension of TPU1 is about 34.45 N m^− 1^, while the TPU2 is about 25.34 N m^− 1^, which is much bigger than the TPU2. The surface tension of THF is 28.8 dyne/cm and the vapor pressure is about 18.9(20 °C), while the surface tension of acetone is 18.8 dyne/cm and the vapor pressure is about 24.64 (20 °C). If the solution vapor pressure is too high, then the solvent will evaporate too quickly and the solution will not be able to make a Taylor cone, while if it is too low, then the fibers will reach the collection plate wet and will merge to form a film. In coaxial spinning, it is usually advantageous to use solvents (or solvent mixtures) with different vapor pressures to avoid fiber collapse [19].

Besides, the solution miscibility between the core and shell is another important factor. As shown in the literature [20], when used the same solvent in the core and shell solution, it enables lower interfacial tension, which is important for the polymer not to precipitate at the fluid interface near the nozzle. As been shown in Table 1, the solvents of CA solution are DMAc and acetone (1/2 volume ratio), which are similar with the solvent of TPU2 and resulted similar interfacial tension between the CA/TPU2 solution interfaces. It also explains the results that the CA/TPU2 fibers can fabricate helical structures more effectively compare with the CA/TPU1 system in Fig. 4. In general, the solvent property will cause a huge change in the spinning solution properties, thus affecting the composite fiber morphology. However, besides the solution property, the polymer material performance also has an important influence on the formation of helical fibers.

### Hydrogen Bonding in Blends

In our previous research, we found that not any polymer component with differential rigidity can form helical fibers, for example, PAN/TPU and PS/TPU system cannot form helical fibers, while Nomex/TPU system could. One of the important reasons is that hydrogen bonds between Nomex/TPU systems help to increase the solution interface interaction.

**Fig. 6:**
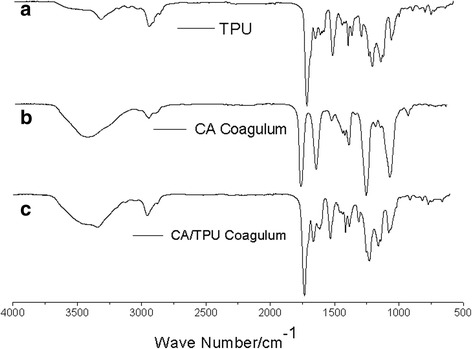
FTIR of the CA/TPU component system including pure polymers and the blends: **a** TPU coagulum, **b** CA coagulum, and **c** CA/TPU coagulum

Figure 6 shows infrared spectra in the range of 500–4000 cm^−1^ of the CA/TPU system. Figure 6a shows a sharp band centered at 1250 cm^−1^ for pure CA coagulum, corresponding to the ether-bonded –O–, which confirmed the CA heterocycle existence. As to the case of the ester group, it shows a strong band centered at 1100 cm^−1^ for pure CA, and at the same time, the interim stretching vibration of carbonyl group –C=O shows a band centered at 1650 cm^−1^. Whereas in the CA/TPU blend, the band 1650 cm^−1^ disappeared and a band centered at 3400 cm^−1^ significantly increased, indicating the formation of a new hydrogen bonding between the –NH in TPU and the oxygen in CA. These data from Fig. 6 suggest that CA is partially miscible with TPU due to the formation of hydrogen bonding between their polymer chains, and the extent of miscibility undoubtedly played an important role in the formation of helical fibers [21].

**Fig. 7:**
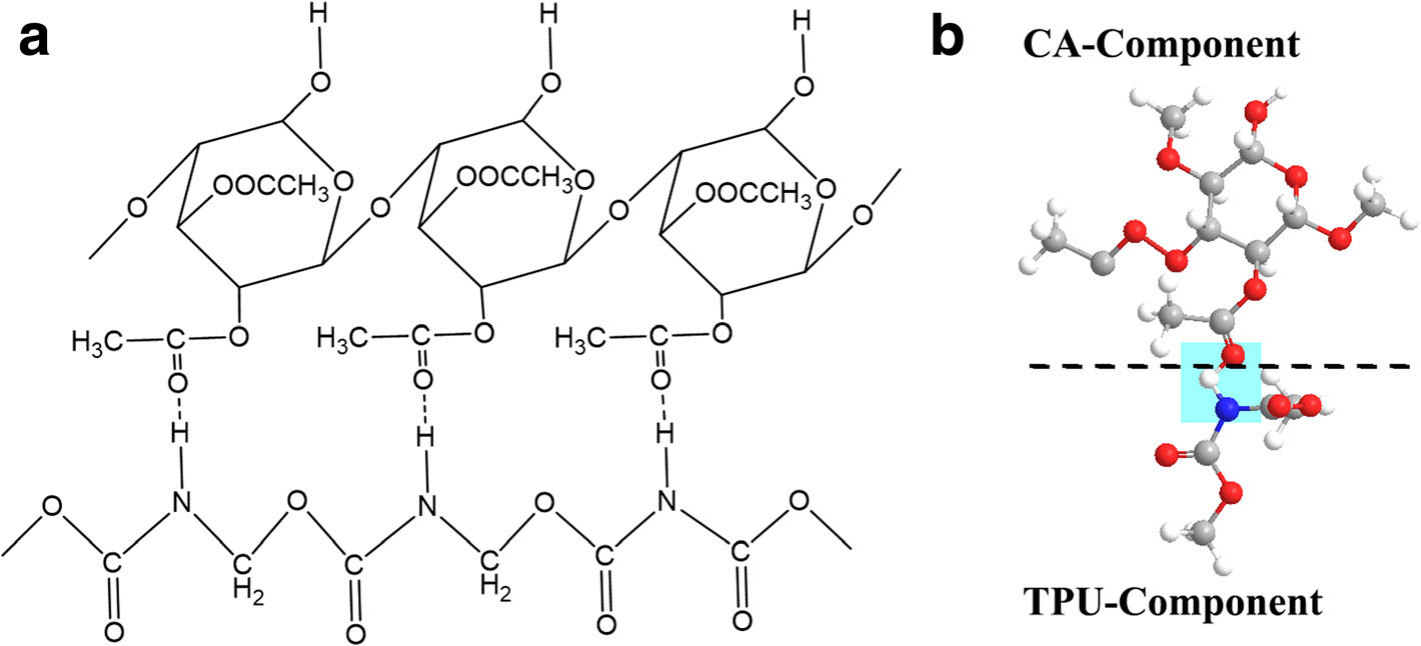
**a**, **b** Hydrogen bonding between polymer chains of CA component and TPU component

The formation of hydrogen bonding between CA and TPU can be further illustrated in Fig. 7. Co-electrospinning involves two polymer solutions which introduce an interface between the two solutions. When stretched by the electric field, a stress, which causes shearing at the interface of the two solutions, is generated. It is expected that the extent of “stress,” considered to be the viscous dragging [22], and in the blends, there is usually necessary to ensure that hydrogen bonding exists between the two base components. As shown in the CA/TPU system, the amide hydrogens –NH in TPU polymer chain are shown in hydrogen bond to the oxygens in CA. It was believed that in the CA/TPU system, hydrogen bonds increase the viscous drag at the interfaces of the composite jets as they are stretched in the electric fields. During the co-electrospinning process, the viscous drag helps the core and shell layer bond together, which is the basis for generating helical bicomponent fibers, because such kind of intermolecular bonding helps to increase the interface interaction between the two layers [23]. Therefore, the CA/TPU system tends to generate helical structures effectively due to the intensified interface interactions.

### Miscibility Behavior in Blends

**Fig. 8:**
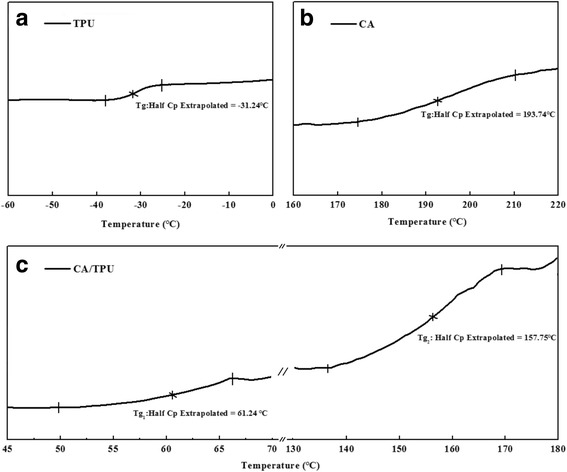
DSC thermograms of the CA/TPU component system including pure polymers and the blends: **a** TPU coagulum, **b** CA coagulum, and **c** CA/TPU blend coagulum

Besides the miscibility in the blends, the longitudinal compressive stress arising from the resiliency of the flexible component (i.e., TPU) and the rigidity of the stiff component (i.e., CA) is fundamental for the formation of helical structures. The glass transition temperature of a polymer, Tg, is an important intrinsic property that influences both the physical and mechanical properties including strength, toughness, and stiffness. Typically, polymers with high chain rigidity have higher Tg [24, 25]. The DSC analysis is one of the convenient methods to determine the polymer glass transition temperature and the miscibility of the polymer blends. Figure 8 shows the DSC thermograms of the TPU, CA, and CA/TPU systems. It can be found that TPU has a Tg of about − 31.24 °C, indicating a quite flexible polymer chain of TPU (Fig. 8a), and CA has a higher Tg (about 193.74 °C) than TPU, indicating the greater chain rigidity of CA. Figure 8c illustrates that in the CA/TPU blend, there are two Tgs (61.24 and 157.75 °C) located between the Tgs of the two individual polymers (− 31.24 °C for pure TPU and 193.74 °C for pure CA), which gives an indication of partial miscibility in blend.

It can be predicted that the more significant of the rigidity differential of the two components, the greater potential for the component system to generate helical structures in co-electrospinning due to the greater interfacial stress between the components. By analyzing the miscibility of the CA/TPU systems, we believe that the partial miscible CA/TPU system tends to generate helical structures due to the intensified interfacial interaction attributed to hydrogen bonding.

## Conclusions

The experimental results show that the CA/TPU2 system could form helical nanofibers effectively because the TPU2 solution enables lower interfacial tension with CA solution. Based on the interfacial interaction induced by the polymer structure and intrinsic properties, we explore the mechanism of CA/TPU helical structures from the three aspects: solution properties, hydrogen bonding, and miscibility behavior of the two solutions. When the solutions are charged, an attractive force between the chloride-ions contained in CA molecules and the free charges on the solution surface lead to a longitudinal interfacial interaction in the CA/TPU system. The large rigidity differential of polymer chains of CA and TPU leads to a large interfacial interaction between them. At the same time, the hydrogen bonds between the polymer chains help to obtain a partial miscible blend of the CA and TPU and consequently increase the interfacial interaction between these two components. This study provides an insight into the mechanism of CA/TPU helical fiber formation and introduces a richer choice of materials for the application of helical fibers.

## Abbreviations

CA: Cellulose acetate
DMAc: N, N-dimethylacetamide
DSC: Differential scanning calorimetry
HSPET: Poly(ethylene glycol terephthalate)
LiCl: Lithium chloride
Nomex: Poly(m-phenylene isophthalamide)
PAN: Polyacrylonitrile
PS: Polystyrene
PTT: Poly(ethylene propanediol terephthalate)
PU: Polyurethane
THF: Tetrahydrofuran
TPU: Thermoplastic polyurethane
TPU1: TPU dissolved in DMAc/THF, 3/1 volume ratio
TPU2: TPU dissolved in DMAc/Acetone, 3/1 volume ratio
FTIR: Fourier transform infrared spectroscopy
